# A risk model of gene signatures for predicting platinum response and survival in ovarian cancer

**DOI:** 10.1186/s13048-022-00969-3

**Published:** 2022-03-31

**Authors:** Siyu Chen, Yong Wu, Simin Wang, Jiangchun Wu, Xiaohua Wu, Zhong Zheng

**Affiliations:** 1 Department of Gynecologic Oncology, Fudan University Shanghai Cancer Center, Shanghai, China; 2 Department of Oncology, Shanghai Medical College, Fudan University, Shanghai, China

**Keywords:** Ovarian cancer, Platinum response, Prognostic model, Biomarkers

## Abstract

**Background:**

Ovarian cancer (OC) is the deadliest tumor in the female reproductive tract. And increased resistance to platinum-based chemotherapy represents the major obstacle in the treatment of OC currently. Robust and accurate gene expression models are crucial tools in distinguishing platinum therapy response and evaluating the prognosis of OC patients.

**Methods:**

In this study, 230 samples from The Cancer Genome Atlas (TCGA) OV dataset were subjected to mRNA expression profiling, single nucleotide polymorphism (SNP), and copy number variation (CNV) analysis comprehensively to screen out the differentially expressed genes (DEGs). An SVM classifier and a prognostic model were constructed using the Random Forest algorithm and LASSO Cox regression model respectively via R. The Gene Expression Omnibus (GEO) database was applied as the validation set.

**Results:**

Forty-eight differentially expressed genes (DEGs) were figured out through integrated analysis of gene expression, single nucleotide polymorphism (SNP), and copy number variation (CNV) data. A 10-gene classifier was constructed which could discriminate platinum-sensitive samples precisely with an AUC of 0.971 in the training set and of 0.926 in the GEO dataset (GSE638855). In addition, 8 optimal genes were further selected to construct the prognostic risk model whose predictions were consistent with the actual survival outcomes in the training cohort (*p* = 9.613e-05) and validated in GSE638855 (*p* = 0.04862). PNLDC1, SLC5A1, and SYNM were then identified as hub genes that were associated with both platinum response status and prognosis, which was further validated by the Fudan University Shanghai cancer center (FUSCC) cohort.

**Conclusion:**

These findings reveal a specific risk model that could serve as effective biomarkers to identify patients’ platinum response status and predict survival outcomes for OC patients. PNLDC1, SLC5A1, and SYNM are the hub genes that may serve as potential biomarkers in OC treatment.

**Supplementary Information:**

The online version contains supplementary material available at 10.1186/s13048-022-00969-3.

## Introduction

Ovarian cancer (OC), the most lethal gynecological cancer, is one of the main causes of cancer-related death among females worldwide [1]. The five-year overall survival rate of epithelial OC patients ranges from 20% at stage IV to 89% at stage I, however, 80% of OC cases can not be diagnosed timely until the tumor has progressed to advanced stages with severe clinical outcomes due to its insidious onset without specific clinical manifestations and the lack of mature early diagnosis methods [2]. Cytoreductive surgery followed by chemotherapy based on platinum or combined with taxanes is the standard treatment for OC [3]. Although most patients with OC show initially highly response to platinum therapy, tumors demonstrate increasing resistance during treatment inevitably. Reportedly, about 70% of patients suffer from tumor relapse a few months after treatment and develop resistance to therapy eventually, no matter primary or secondary resistance, representing the major challenge in OC treatment [4, 5]. Identification of nonresponders is an important step toward greater life expectancy for OC patients [6]. Meanwhile, the specific biomarkers predicting platinum therapy response remain obscure, therefore, it is of vital importance to figure out the potential indicators, which could aid clinical decisions and improve prognosis.

Nowadays, the rapid development of next-generation sequencing (NGS) has revolutionized and renewed how we comprehend cancer treatment and promoted the progress of precision medicine [7]. Increasing evidence has authenticated that molecular biomarkers contribute to the prognosis evaluation and prediction of tumors [8]. Besides, researchers found that rather than conventional single-gene biomarkers, gene signatures containing several genes can provide stronger evidence to prognosis and survival [9]. For example, based on the public database, a six-gene model (TGFBI, SFRP1, COL16A1, THY1, PPIB, BGN) was built and serves as an independent prognostic biomarker for overall survival [10]. Bi et al. identified eight glycolysis-related prognostic genes that effectively predicted survival in ovarian cancer [11]; A tumor mutation burden (TMB) associated immune risk score signature was built by Cui et al. for TMB and prognosis evaluation [12]; Salinas et al. applied SNP data from TCGA to find SNPs associated with chemo-response in ovarian cancer [13]; And another study developed and validated an immune-related gene signature that was significantly associated with survival [14]. Despite encouraging developments, no biomarkers for the prediction of response to therapy and prognosis are applied into clinical practice yet.

**Fig. 1:**
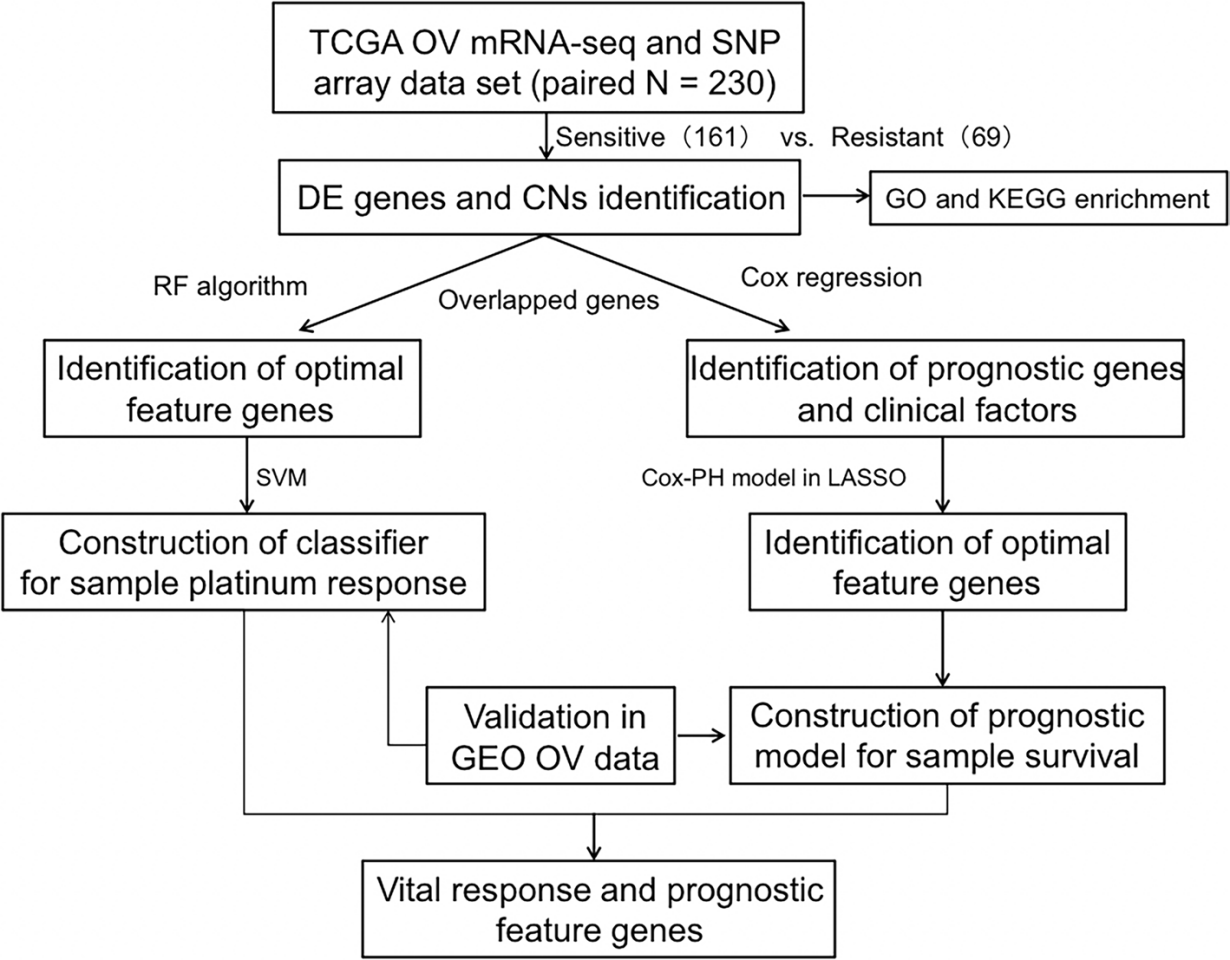
Flowchart of this study

In this study, through bioinformatics data analysis, we integrated the gene expression profiles of the transcriptome, single nucleotide polymorphism (SNP), and copy number variation (CNV) to identify differentially expressed genes (DEGs) firstly. Then a support vector machine classifier was constructed to distinguish patients’ responses to platinum therapy. Next, combined with L1-LASSO and Cox-Proportional Hazards regression, we constructed a prognostic risk model based on 8 optimum genes to predict prognosis which could mirror the prognosis related to platinum response status as well. Finally, after the intersection of the classifier and prognostic model, PNLDC1, SLC5A1, and SYNM were identified as the hub genes related to both platinum response and prognosis, which was further verified by IHC analysis. The flowchart of this study was displayed in Fig. 1. In summary, the comprehensive analysis of gene expression level, SNP and CNV in our study could provide more accurate and robust molecular markers for diagnosis, prediction and bring new insights into clinical treatment strategies for OC patients.

## Materials and methods

### OC datasets extraction

The OC datasets used in this study were derived from TCGA and GEO databases. mRNA-seq expression profile data (platform: Illumina HiSeq 2000 RNA Sequencing) and SNP, CNV information (platform: Affymetrix Genome-Wide Human SNP Array 6.0) were downloaded from TCGA. Collectively, 419 OC tumor tissue samples with expression profiles and 481 samples with SNP information were included. After barcode matching, 230 OC samples possessing clinical platinum response status were obtained, comprised of 69 resistant and 161 sensitive samples respectively, which serve as our training dataset. As for validation dataset, the gene expression profiles of GSE63885(*n* = 101, platform: GPL570 [HG-U133_Plus_2] Affymetrix Human Genome U133 Plus 2.0 Array) from the GEO database (https://www.ncbi.nlm.nih.gov/geo/) was acquired, containing a total of 75 samples having platinum response status, consisting of 34 resistant and 41sensitive samples, respectively. Both the expression profiles and clinical characteristics can be obtained publicly, so there was no need to acquire ethics committee approval. The abovementioned data were displayed in Table 1.

**Table 1:** Clinicopathological characteristics of patients with OC in this study

Variables	Training set(***N*** = 230)			Validation set(***N*** = 75)		
	Resistance (***N*** = 69)	Sensitive (***N*** = 161)	***p*** value	Resistance (***N*** = 34)	Sensitive (***N*** = 41)	***p*** value
Age (years, mean ± sd)	61.77 ± 11.47	58.55 ± 11.32	0.0524^a^	–	–	–
Neoplasm subdivision (Bilateral/Left/Right/−)	51/7/7/4	109/27/14/11	0.4066^b^	–	–	–
Stage (II / III / IV)	1/60/8	10/127/24	0.2167^b^	–	–	–
Histologic grade (G2/G3/G4/−)	7/60/1/1	25/133/0/3	0.3213^b^	–	–	–
Lymphatic invasion (Yes/No/−)	17/7/45	35/30/96	0.282^b^	–	–	–
Recurrence (Yes/No)	61/8	122/5/34	0.06702^b^	–	–	–
Death (Dead/Alive/−)	58/11	82/79	1.674E-6^b^	33/1	33/3/5	0.6145^b^
Overall survival (months, mean ± sd)	29.71 ± 14.34	48.59 ± 25.33	1.342E-11^a^	27.01 ± 14.51	51.82 ± 27.97	1.915E-5^a^

Notes: “-”:not know; ^a^ T-test; ^b^ Fisher test

### Data preprocessing

For the original gene expression profile FPKM data downloaded from TCGA, preprocessCore Version 1.40.0 [15] (http://bioconductor.org/packages/release/bioc/html/preprocessCore.html) in R was used to perform standardization based on the quantiles algorithm. For the SNP 6.0 chip data, PICNIC software [16] (ftp://ftp.sanger.ac.uk/pub/cancer) was applied to convert and process the data of CEL format to obtain CN segment data. (The segment data indicated the copy value in the detection region. Usually, the segment value of the diploid was 0, implying there was no copy number variation, and the other non-zero signal indicated the region was missing or amplificated). The human gene annotation file (Release 27 (GRCh38.p10)) from the GENCODE database (http://www.gencodegenes.org/releases/current.html) was extracted and gene annotation was employed. And at the same time, the oligo package Version 1.42.0 [17] (http://www.bioconductor.org/packages/release/bioc/html) was used to convert to the original data of the GSE63885 data set, fill the missing data (median method), conduct background correction (MAS method) and data standardization (quantiles).

### DEGs identification

After normalization, in the light of platinum response status, we divide the patients into two groups: chemotherapy-sensitive (*n* = 161) and chemotherapy-resistant (*n* = 69). Differently expressed genes and genes with different CN signals were selected based on the ‘limma’ package [18]. Significant DEGs were defined as those with adjusted *P* < 0 .05 and |log FC| ≥1. The overlapping genes, that is, genes with significant differences in both expression level and CN signal value between the resistant and sensitive groups were further screened out. Next, only genes containing CN variant sites were retained (variant types include SNP, INS, DEL, etc.). We used DAVID version6.8 [19] (https://david.ncifcrf.gov/) to analyze the DEGs for molecular function (MF), cellular component (CC), biology procedure (BP) enrichment by studying the Gene Ontology (GO) terms. And the Kyoto Encyclopedia of Genes and Genomes (KEGG) pathway enrichment of DEGs was carried out as well. A *p*-value < 0.05 was set as the threshold for significant enrichment. And these genes with significantly different CNV expressing were used for further analysis.

### Construction and validation of the classifier

#### Selection of best representative gene features using Random Forest

We identified the best combination of representative genes using a Random Forest machine learning method. The Random Forest method is an ensemble algorithm comprised of a series of decision trees. Each tree randomly selects several features in the sample zone to make a prediction. These predictions will be aggregated, and the final prediction will be decided using a voting method which refers to the category having the highest votes.

We implemented the Random Forest model using the randomForest R package Version 4.6–12 [20] (https://cran.r-project.org/web/packages/randomForest/index.html). The model was built on expression levels of genes identified in TCGA samples. Details of the algorithm were as follows:

I. We randomly sampled k samples from TCGA samples with replacement using a bootstrap method to construct k regression trees for classification. The unselected samples in each round constructed k out-of-bag (OOB) sets (k was iteratively set from 1 to the total number of samples N).
II. For a total number of n features, we randomly selected m features at each splitting node of each tree and calculated the predicting power of each feature. We then exploited the most powerful feature to assign samples at that node. N was set from 1 to the total number of variables and m was set to the secondary square root of the total number of variables.
III. We let each tree grow to the maximum without any pruning.
IV. We aggregated all decision trees to construct a Random Forest (RF) model. The RF model adopted a voting method that defined the category with the highest votes as the final classification.
V. We evaluated the RF model using the OOB error rate and features of the model with the lowest OOB error rate were selected as the optimal combination.

#### Development and validation of the SVM classifier

We used the e1071 R package (https://cran.r-project.org/web/packages/e1071) to develop an SVM model [21] (Support Vector Machine). SVM is a supervised machine learning method for classification. Using representative features of each sample, the model predicts the possibility belonging to a certain category to implement classification. We developed the SVM model on the TCGA training set, using “Sigmoid” as the Kernel and the optimal signature genes as features. The parameters were selected by 100-fold cross-validation. To test the performance of our model, we measured five indicators on a validation set GSE63885, including sensitivity, specificity, positive prediction value (PPV), negative prediction value (NPV) and area under curves (AUC) of the receiver operating characteristic curve (ROC) [22]. Calculation methods of these indicators are listed below:

**Table:** 

		Observed	
		positive	negative
Predicted	positive	A	B
	negative	C	D

Sensitivity = A/(A + C); Specificity = D/(B + D); PPV (Positive Predictive Value) = A/(A + B); NPV (Negative Predictive Value) = D/(D + C)

### Construction of the prognostic risk model

#### Selection of prognostic genes and clinical factors

We performed univariate Cox-PH (proportional hazards) regression model to select prognostic genes and clinical factors. Based on the expression levels of genes in 2.3 of TCGA samples, the model was built by the survival R package (Version 2.41–1) [22]. Prognostic genes and clinical factors were identified using *P* < 0.05 as the threshold (log-rank test).

#### Selection of the optimal genes

Based on expression levels of the prognostic genes identified prior, we implemented L1-Regularized Cox-PH regression analysis on TCGA samples to select the optimal combination of these genes [23]. The model was developed using the penalized R package (Version0.9–50, http://bioconductor.org/packages/penalized/) [24]. The “lambda” parameter was identified by the 1000 cross-validation likelihood (cvl) method.

#### Development of a Cox-PH model using optimal genes

We constructed a prognosis score (PS) using coefficients of optimal genes from the Cox-PH regression model. Using the median of PS as the threshold, we separate training samples into high and low-risk groups. The prognostic value of PS was then evaluated by the Kaplan-Meier survival curve [25] using log-rank test in the training set and then validated in the GSE63885 dataset.

### Hub genes screening and validation

#### Selection of the hub genes related to platinum therapy and prognosis

Further comparison between feature genes comprised in SVM classifier and included in the prognostic risk prediction model, crucial genes were screened out.

#### Validation via immunohistochemistry

A total of 80 OC patients from FUSCC who received platinum-based chemotherapy after surgery were selected randomly, and tissue microarrays composed the representative cores from each specimen. Immunohistochemistry proceeded as described before [26]. In brief, specimens were incubated first with an anti- PNLDC1 antibody (1:2000, Proteintech, China), anti-SLC5A1 antibody (1:1000, Abcam, UK) or anti-SYNM antibody (1:200, Proteintech, China) overnight at 4 °C and then with a biotinylated secondary antibody (1:100, goat anti-rabbit IgG) for 30 min at 37 °C. A well-established IRS was then used to calculate the protein expression level of these three hub genes [27]. Firstly, the staining intensity (SI) was scored using a 4-point scale from 0 to 3, with 0 if there was no staining. For weak, moderate, and strong staining, the scores were 1, 2 and 3, respectively. Secondly, the percentage of positive cells was scored into five categories: no staining, 1–10, 11–50, 51–80, 81–100 percentage positive cells. And the scores were 0, 1, 2, 3 and 4, respectively. An IRS was calculated by multiplying the percentage of hub genes by the SI score, resulting in a scale from 0 to 12. The IRS was divided into four groups: 0 (IRS 0–1), 1 (IRS 2–3), 2 (IRS 4–8) and 3 (IRS 8–12). Then, 0 and 1 were stratified into low expression group and 2 and 3 into high expression group and performed survival analyses. The expression of each hub gene was quantified by using an Image-Pro Plus Image Analysis Software and the IOD (Integral optic density) was measured as reported previously [28]. To find the optimal cut-off points, the X-tile program was used [29].

### Statistical analyses

All statistical analyses in this study were performed by R (version 3.4.1). The statistical significance threshold was set at 0.05 if not explicitly mentioned. In our study, progression-free survival (PFS) is defined as the time from operation to relapse or progression, whichever occurred first. And patients were divided into platinum-sensitive and platinum-resistant subgroups according to the platinum-free interval (PFI). PFI is defined as the time from the end of the first chemotherapy course to disease recurrence, and PFI > 6 months was regarded as platinum-sensitive, whereas PFI < 6 months was the platinum-resistant group.

## Results

### Data pre-processing and DEGs screening

**Fig. 2:**
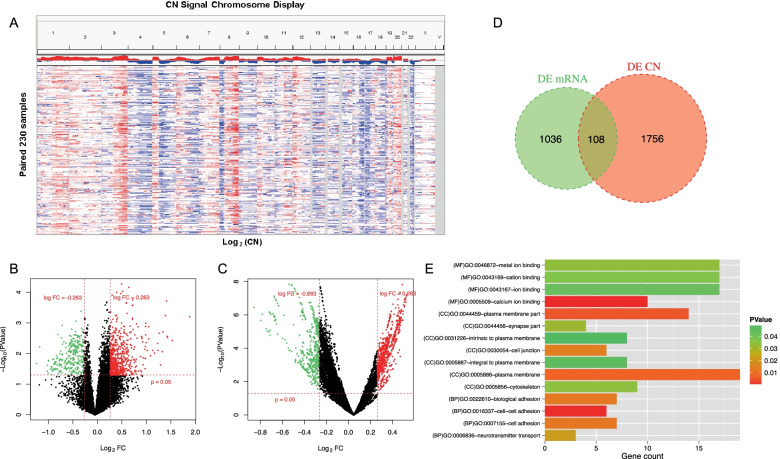
Identification of DEGs. **A** The CN signal of the TCGA samples. The horizontal axis represents the detection area on each chromosome, the vertical axis represents the 230 ovarian cancer samples included in the analysis. 1–22 and X, Y indicates the chromosome number, and blue indicates log2 (CN) < 0, while red indicates log2 (CN) level > 0. **B** Volcano plots of the DEGs in gene expression. **C** Volcano plots of the DEGs in CN signals. **D** The Venn diagram of the DEGs and 108 genes as overlapping genes in both expression and CN signal levels. **E** Gene Ontology (GO) functional enrichment analysis of the 48 DEGs in the biological process subsection of GO (BP); molecular function subsection of GO (MF); a cellular component subsection of GO (CC)

The gene expression profiles obtained from TCGA and GEO datasets were firstly normalized and the box diagram before and after standardization was shown in Supplementary Figure 1. Concerning the data of CN signal, gene annotation was performed, followed by depicting the distribution of chromosomes. And we found that, in different samples, but the same sites, the CN signals distributed similarly (Fig. 2A). Via Limma package in R, 1144 DEGs differently expressed (524 downregulated and 620 upregulated) and 1864 DEGs with diversified CN signals (727 downregulated and 1137 upregulated) between platinum-sensitive and the resistant group were obtained from the TCGA database (Fig. 2B and C, Supplementary Table 1).

Further analysis uncovered 108 genes as overlapping genes in both expression and CN signal levels (Fig. 2D, Supplementary Table 2). Integrated with SNP information, we found 48 genes had variant sites, including 94 SNP,1 INS, and 1 DEL (Supplementary Table 3), indicating they were differentially expressed and had diversified CNV signals between platinum-resistant and sensitive groups simultaneously. To reveal the biological functions of the 48 DEGs, the GO and KEGG enrichment analyses conducted by DAVID were employed (Fig. 2E). Regarding biological process (BP), the GO analysis results showed that the intersecting DEGs were mainly enriched in terms related to cell adhesion (Supplementary Table 4). As for KEGG pathway analysis, the DEGs were enriched in Cell adhesion molecules (Table 2).

**Table 2:** DEGs significantly related KEGG pathways

Term	Count	*P* Value	Genes
hsa04514: Cell adhesion molecules (CAMs)	2	0.021093	CD274, NLGN1
hsa04020: Calcium signaling pathway	2	0.027187	NOS2, CACNA1C
hsa05200: Pathways in cancer	2	0.045151	NOS2, MMP1

### Construction and validation of the classifier

**Fig. 3:**
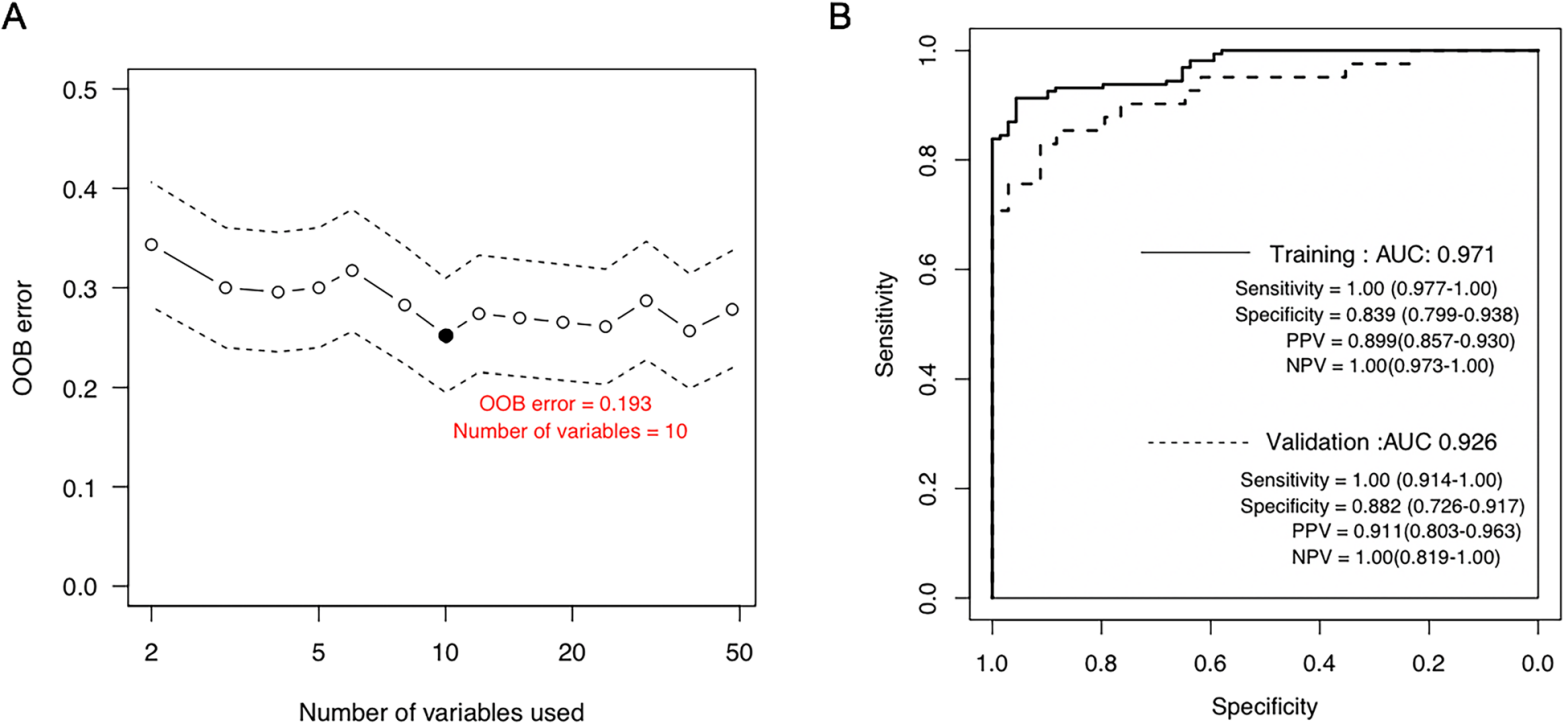
Construction and evaluation of the SVM classifier. **A** OOB error calculated by the RandomForest algorithm and 10 genes were selected optimally when the OOB error is the smallest. **B** The receiver operating characteristic (ROC) curve (area under the curve (AUC) of the TCGA training set (solid line) and GSE63885 validation set (dotted line)

To extract the most representative and feature genes in these 48 genes, the RandomForest algorithm was performed. And 10 genes were figured out optimally when the OOB error is minimum (Fig. 3A). And we found that the CNV of the 10 genes were all SNPs, comprising one known SNP site in CD209 and 9 unreported SNP sites (Table 3).

**Table 3:** 10 optimal feature genes contained in the SVM classifier

Gene	DE mRNA		DE CN		Variant								
	logFC	*p*.value	logFC	*p*.value	Chrom	Variant Position	Type	Variant Classification	Amino acids	Codons	Reference Allele	Tumor Allele	dbSNP_RS
CD209	0.358543	0.0142	−0.42485	0.012717	19	7,807,996	SNP	Missense	R	Agg/Cgg	C	T	rs11465393
CD274	0.437529	0.00976	0.460916	0.002227	9	5,462,907	SNP	Missense	G/V	gGg/gTg	G	T	novel
HIST1H3I	−0.43754	0.00255	0.30903	0.010138	6	27,839,796	SNP	Missense	P/LX	cca/cTca	A	G	novel
HIST1H4L	−0.43335	0.0143	0.30903	0.010138	6	27,841,216	SNP	Missense	T	acA/acG	C	T	novel
NLGN1	1.019701	0.00143	0.351201	0.004621	3	173,525,589	SNP	Missense	T/M	aCg/aTg	G	C	novel
NTRK3	−0.36699	0.0247	0.295746	0.007258	15	88,576,170	SNP	Silent	L	Ctg/Ttg	C	G	novel
						88,483,904	SNP	Nonsense	T	acG/acC	C	A	novel
						88,522,597	SNP	Intron	R/W	Cgg/Tgg	G	A	novel
						88,423,602	SNP	Missense	T	acG/acA	G	A	novel
						88,420,205	SNP	Missense	D/X	Gat/at	C	A	novel
						88,476,380	SNP	Missense	Y	taC/taT	A	T	novel
PNLDC1	−0.85134	0.000718	−0.34754	0.003742	6	160,240,313	SNP	Silent	I/M	atC/atG	G	C	novel
SLC22A3	−0.51215	0.0225	−0.30286	0.005976	6	160,872,083	SNP	Missense	Y/*	taC/taG	C	T	novel
SLC5A1	0.298462	0.00642	0.441319	0.001623	22	32,495,226	SNP	Nonsense	V	gtC/gtT	C	G	novel
SYNM	0.558712	0.00288	0.448385	0.001034	15	99,672,483	SNP	Silent	T/R	aCg/aGg	A	G	novel
						99,669,768	SNP	Silent	T	acC/acT	G	A	novel
						99,671,917	SNP	Missense	R	cgG/cgC	G	C	novel
						99,672,905	SNP	Missense	T/N	aCc/aAc	C	G	novel
						99,653,864	SNP	Silent	D/E	gaT/gaA	C	T	novel
						99,672,085	SNP	Missense	Y/H	Tac/Cac	G	T	novel

Based on expression profiles of the above 10 feature genes in the TCGA dataset, we constructed an SVM classifier to determine the platinum-sensitive and resistant samples. It was able to accurately distinguish 212 out of 230 samples (161 sensitivity vs. 51 resistance), with a precision rate of 92.17% and an average AUC of 0.971 (Fig. 3B, solid line). The sensitivity and specificity were 1 and 0.839, respectively, and the PPV and NPV are 0.899 and 1 as well. To further verify and evaluate the predictive effects of this model, GSE63885 was used as an independent external validation dataset. The result of the validation cohort showed that 71 samples (41sensitivity vs. 30 resistance) out of 75 samples could be discriminated precisely, and the accuracy rate was 94.76% with an AUC of 0.926 (Fig. 3B, dotted line). The sensitivity, specificity, PPV, and NPV were 1, 0.882, 0.899, and 1, respectively. To sum up, this model could accurately classify the drug-sensitive samples and moderately found the drug-resistant patients, indicating these 10 genes had strong correlations with drug sensitivity. The gene profiles of 10 feature genes in the TCGA and GSE638855 datasets were displayed in Supplementary Table 5.

### Construction and validation of the prognostic risk model

#### Univariate cox regression analysis

**Fig. 4:**
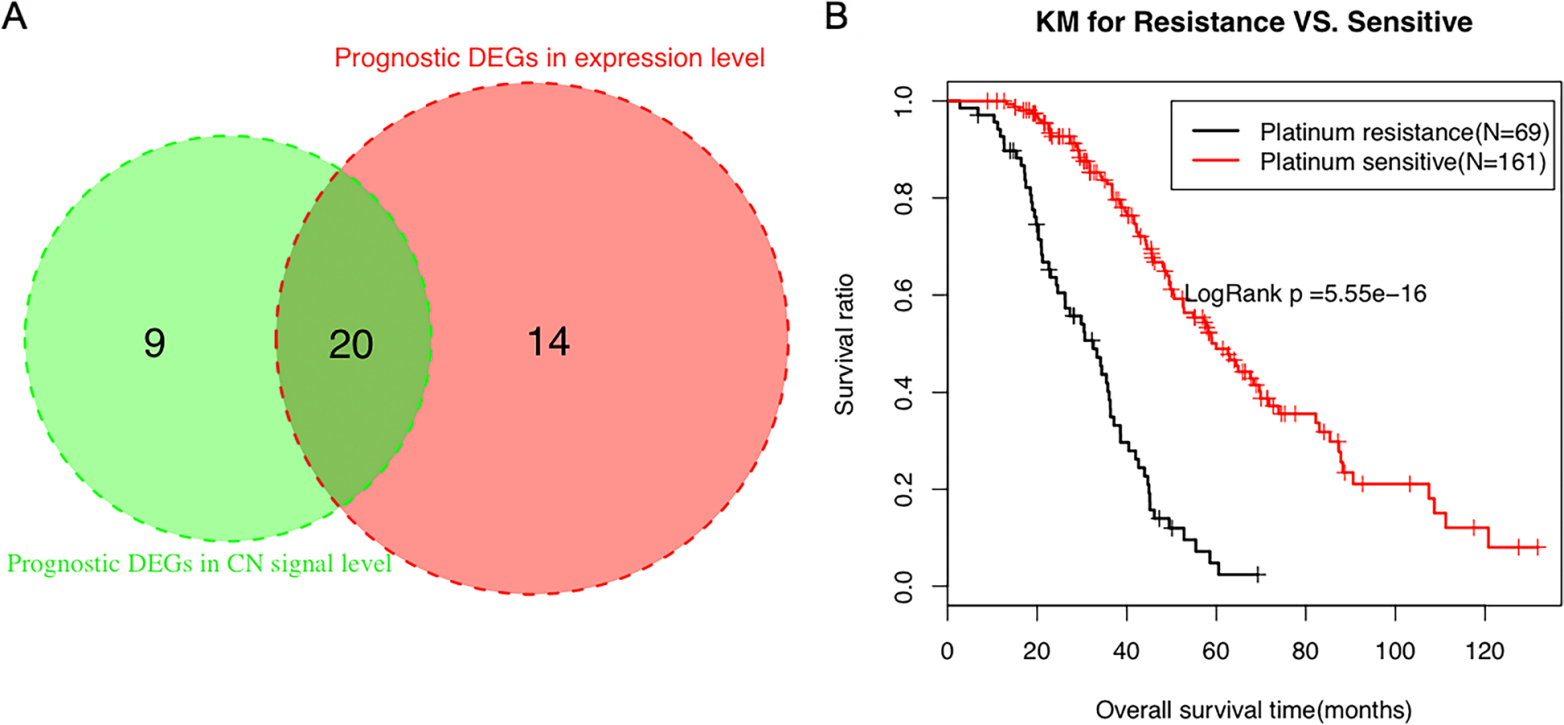
Survival analysis of the DEGs and clinical factors. **A** The Venn diagram of DEGs related to prognosis. **B** The K-M curve of the overall survival of the patients with different platinum response statuses

Combined with the clinical information, the overlapping 48 DEGs were filtered via univariate cox regression analysis in the TCGA training cohort to acquire genes significantly related to prognosis. Consequently, 34 genes were differentially expressed and 29 genes with different CN signals were obtained separately (Supplementary Table 6). After the intersection, 20 crossed genes were left (Fig. 4A). Meanwhile, the clinicopathological factors related to prognosis identified by univariate analysis were merely platinum response status (Table 4). And a conspicuous OS difference was noted between sensitive and resistant Kaplan-Meier curve (HR = 0.22, *p* = 5.55e-16), reflecting a better survival in the platinum-sensitive group (Fig. 4B). It also verified that the genes we screened were indeed related to the platinum response status to some extent.

**Table 4:** Univariate cox regression analysis of clinicopathological characteristics

Variables	Univariate analysis		
	HR	95% CI	*P* value
Age	1.008	0.9929–1.024	0.2972
Platinum response status (Sensitive/Resistant)	0.2168	0.1498–0.3137	5.55E-16
Neoplasm subdivision (Bilateral/Left/Right)	0.8438	0.6318–1.127	0.249
Stage (II / III / IV)	1.052	0.7266–1.524	0.787
Lymphatic invasion (Yes/No)	0.9587	0.5293–1.736	0.889
Histologic grade(G1-G2/G3-G4)	1.272	0.8054–2.009	0.3015
Recurrence (Yes/No)	0.6948	0.3519–1.372	0.2913

#### Selection of the optimal genes

**Fig. 5:**
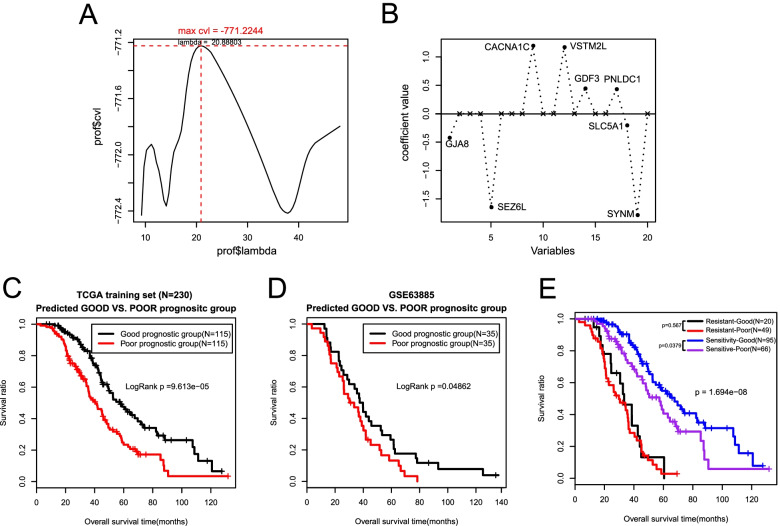
Construction and validation of the prognostic risk model. **A**. Cross-validation likelihood filters the lambda parameter (20.88803) when cvl takes the maximum value (− 771.2244). **B** Based on the L1-penalized regularized regression algorithm, the optimal prognostic gene coefficient distribution line for Cox-PH model screening. **C** Prognostic prediction by the prognostic risk model in the TCGA training dataset. **D** Prognostic prediction by the prognostic risk model in the GSE63885 validation set. **E** The correlation between the K-M curve of the platinum response status and the prognostic model prediction

According to the intersectional 20 genes related to prognosis, the Cox-PH model based on the L1-penalized regularized regression algorithm was exploited to further select the optimal genes. The maximum value of cvl − 771.2244 was obtained when the lambda value is 20.88803 after 1000 cycles of cvl algorithm calculations (Fig. 5A). Under this Circumstance, 8 optimum genes were received (Table 5) and the gene prognostic coefficients are shown in Fig. 5B.

**Table 5:** Optimal prognostic-related genes used to construct the prognostic risk model

Gene	coef	Hazard Ratio	95%CI	*p* value
GJA8	−0.42542	0.8324	0.5970–1.1605	0.00068
PNLDC1	0.430375	1.2266	0.9523–1.5799	0.00225
SLC5A1	−0.20707	0.9885	0.8578–1.1391	0.01138
VSTM2L	1.169891	1.1904	1.0645–1.3313	0.01152
CACNA1C	1.195075	2.2235	1.4023–3.5256	0.027923
SEZ6L	−1.64918	0.6669	0.4555–0.9763	0.03024
GDF3	0.442726	1.4139	1.0336–1.9340	0.03722
SYNM	−1.78725	0.5982	0.4016–0.8912	0.04873

#### Construction and validation of the prognostic risk model

Based on the Cox-PH prognostic coefficients of the 8 optimized genes, a risk model was constructed by the following formula: Prognosis score (PS) = (− 0.42542) × ExpGJA8+ (0.430375) × ExpPNLDC1 + (− 0.20707) × Exp SLC5A1 + (1.169891) × ExpVSTM2L +(1.195075) × Exp CACNA1C +(− 1.64918) × ExpSEZ6L+(0.442726) × ExpGDF3 + (− 1.78725) × Exp SYNM.

To validate the survival-predicting performance of the model, the prognostic score (PS) of each sample was calculated and the median PS was applied as the threshold to subdivide the training cohort into a high-risk group (HRG) and a low-risk group (LRG). First, in the training set, the correlation between the model’s predictions and the actual prognosis was evaluated through the Kaplan-Meier curve. We discovered that LRG had a longer median OS time than HRG. In detail, the median OS of HRG (115 samples) was 38.56 ± 21.31 months, while the average OS of the LRG (115 samples) was 47.30 ± 26.11 months (Fig. 5C). And the correlation between the groupings predicted by the model and the actual survival outcome was significant and consistent (*p* = 9.613e-05). Concurrently, the results of the validation set GSE63885 saw identical results, showing that the average survival time of the HRG (35 samples) was 33.78 ± 18.33 months, whereas the LRG (35 samples) had a longer median OS of 46.11 ± 30.49 months (Fig. 5D). Model predictions and actual results had a significant correlation (*p* = 0.04862). The detailed survival information of the TCGA and GSE63885 datasets and the PS information of the samples were shown in Supplementary Table 7.

Additionally, to further clarify the correlation between the prediction of the prognostic model and the platinum response status, subgroup survival analysis dividing into the sensitive and resistant groups was carried out (Fig. 5E). On the whole, the prognosis predicted by the prognostic model is significantly correlated with the prognosis that depends on the platinum response state (*p* = 1.694e-08). Specifically, in the sensitive subgroup, the prognosis prediction of the model was remarkably correlated with the actual survival. It is supposed that after being determined by the SVM classifier, patients who identified as the sensitive group could accept platinum-based chemotherapy continually and the prognostic model could foresee the prognosis accurately. Conversely, patients who were considered to be resistant could alter and optimize therapeutic strategies as early as possible. In a nutshell, our model had an important role in different ways for the two groups which helped with the clinical decision to some degree.

### Identification of hub genes related to platinum response and prognosis

**Fig. 6:**
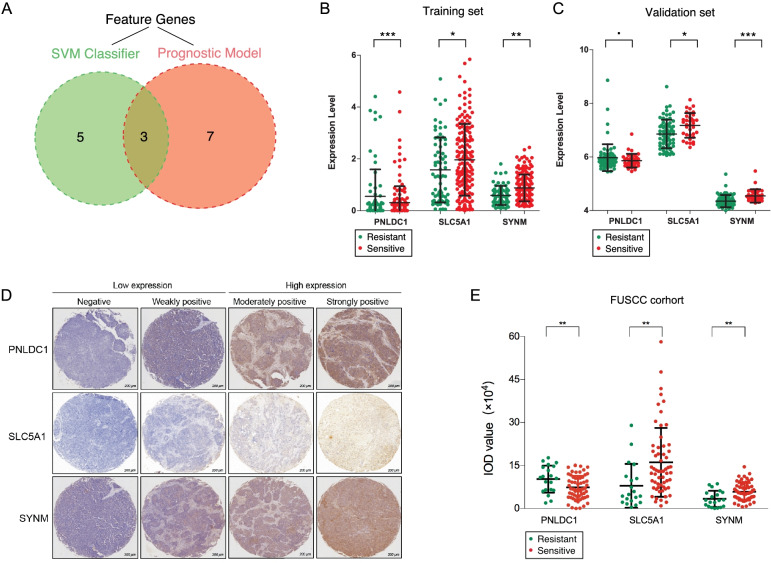
Screening and validation of the hub genes. **A** The intersection of the genes between the SVM classifier and the prognostic risk model. **B** The expression of PNLDC1, SLC5A1, and SYNM in the training TCGA database. **C** The expression of PNLDC1, SLC5A1, and SYNM in the validation set GSE63885. **D** Representative immunohistochemistry images of PNLDC1, SLC5A1, and SYNM. **E** The expression of PNLDC1, SLC5A1, and SYNM in the FUSCC cohort

To find hub genes, genes used to establish the SVM classifier and the prognostic model were consolidated, and 3 intersecting genes named PNLDC1, SLC5A1, and SYNM were obtained (Fig. 6A). And we found that, both in training and validation sets, PNLDC1 was down-regulated in the sensitive group while SLC5A1 and SYNM were up-regulated (Fig. 6B and C; Supplementary Table 8), hence we assumed that these 3 genes reflect both platinum response status and prognosis, and their CNV information is shown in Table 6.

**Table 6:** The CNV information of the 3 hub genes

Gene	Variat								
	Chrom	Variant Position	Type	Variant Classification	Amino acids	Codons	Reference Allele	Tumor Allele	dbSNP_RS
PNLDC1	6	160,240,313	SNP	Silent	I/M	atC/atG	G	C	novel
SLC5A1	22	32,495,226	SNP	Nonsense	V	gtC/gtT	C	G	novel
SYNM	15	99,672,483	SNP	Silent	T/R	aCg/aGg	A	G	novel
		99,669,768	SNP	Silent	T	acC/acT	G	A	novel
		99,671,917	SNP	Missense	R	cgG/cgC	G	C	novel
		99,672,905	SNP	Missense	T/N	aCc/aAc	C	G	novel
		99,653,864	SNP	Silent	D/E	gaT/gaA	C	T	novel
		99,672,085	SNP	Missense	Y/H	Tac/Cac	G	T	novel

**Fig. 7:**
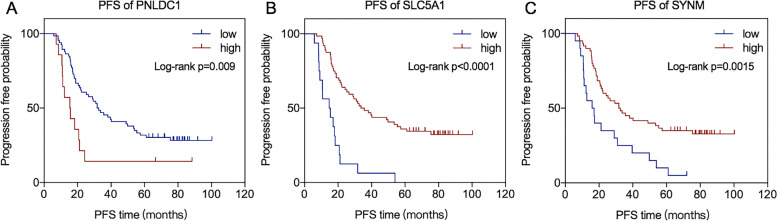
Survival analysis of the hub genes. **A** PFS analysis of PNLDC1. **B** PFS analysis of SLC5A1. **C** PFS analysis of SYNM

To confirm their relationships with platinum response status and prognosis, IHC of 80 patients from the FUSCC cohort was applied (Fig. 6D). And in the FUSCC cohort, 20 patients were resistant to platinum-based therapy and the other 60 were in the sensitive group (Supplementary Table 9). In line with our former findings, the intensity and quantity of PNLDC1’s expression were remarkably higher in the resistant group (*p* = 0.0096), while SLC5A1 (*p* = 0.0058) and SYNM (*p* = 0.0022) were significantly amplified in the sensitive patients (Fig. 6E). Since the deficient number of events regarding the OS analysis, PFS was analyzed instead. X-tile was exploited to find the best cut-off points with minimum *p* values. The K-M curve uncovered that those patients with high expression of PNLDC1 had shorter PFS (Log-rank *p* = 0.009, Fig. 7A). Conversely, survival analysis demonstrated that the SLC5A1 and SYNM high group showed adverse outcomes (Log-rank *p* < 0.001, Fig. 7B; Log-rank *p* = 0.0015, Fig. 7C). These results further supported that PNLDC1, SLC5A1 and SYNM were the hub genes associated with the platinum response status and prognosis indicating they could be used as potential biomarkers in clinical practice.

## Discussion

Nowadays, the common treatment regimen for OC consists of tumor debulking, followed by administration of platinum-based chemotherapy [30], however, resistance to platinum therapy limits therapeutic options, and makes platinum-resistant patients the most challenging to treat. Apart from PFI, regarding as a predictive factor to subsequent platinum therapy [31], biomarkers reflecting platinum response status are urgently needed. Therefore, to begin with, the 230 samples in the TCGA database were stratified into the sensitive and resistant groups, and through comprehensive analysis from 3 aspects, including gene expression level, CN signal, and SNP data, we obtained 48 overlapping DEGs. Considering a burgeoning number of researches concerning gene signature of tumors show up, which was supported by the development of RNA-sequencing and microarray, as well as available public databases [14, 32]. We constructed a classifier via the RandomForest algorithm aiming at dividing patients into sensitivity and resistance groups. The classifier comprised of 10 genes (CD209, CD274, HIST1H3I, HIST1H4L, NLGN1, NTRK3, PNLDC1, SLC22A3, SLC5A1 and SYNM), and its validation in the GEO dataset showed a satisfying consistency, which indicating the classifier could differentiate the sensitive group accurately and aid the resistant patients to receive other effective therapy as soon as possible.

Liu et al. used the TCGA dataset to validate a seven genes-based model which can predict the survival of FIGO stage IIIc serous ovarian carcinoma (HG3cSOC) and served as a valuable marker for the response to platinum-based chemotherapy [33], however, they chose HG3cSOC to analysis and confined to gene expression analysis only; Zhao et al. identified AGGF1 and MFAP4 as potential predictors of primary platinum-based chemoresistance [34], nonetheless, they just focused on the gene expression level and lack of experimental-level validation, such as immunohistochemistry; Dugo et al. mainly focused on HGSOC patients who received complete cytoreduction (R0) and analyzed focal copy number alterations [35]; A qualitative transcriptional signature for predicting recurrence risk for high-grade serous ovarian cancer patients was constructed by Liu et al. [36]; Salinas et al. found 19 SNPs were associated with chemo-response [13]. Although these studies have similarities to ours, we conducted a comprehensive analysis from diversified levels to figure out the most robust markers indicating platinum treatment response and prognosis.

Detailedly, the current treatment of OC could prolong the interval between recurrences but does not benefit overall survival [37]. In the study, via univariate Cox regression analyses, genes and clinicopathologic parameters related to prognosis were obtained. And we found that sensitivity to platinum-based therapy was the only clinical factor that contribute to better survival. To make survival predictions, 8 optimum genes were applied to establish the prognostic model, and we found that upregulation of PNLDC1, VSTM2L, CACNA1C, and GDF3 were related to worse clinical outcomes, however, high expressions of GJA8, SEZ6L, SLC5A1, and SYNM were associated with better prognoses. Specifically, PNLDC1, a PARN-like 3′-to-5′ exonuclease located at the membrane of the mitochondria in a mouse, is critical to the processing of piRNAs and PNLDC1 disrupted in mice would lead to azoospermia and male infertility ultimately [38, 39]. Former studies pointed that PNLDC1 was related to survival in CRC patients [40], and PNLDC1 expressed higher in normal colorectal tissues than in cancer tissues [41]. VSTM2L could induce adverse survival outcomes in rectal cancer [42]. CACNA1C, as a voltage-gated calcium channel, is up-regulated in brain tumors, leukemia, breast cancer, and other tumors [43] and plays as an oncogene in OC tumors [44]. GDF3 is widely accepted as a pluripotency marker and expressed in several cancer types such as breast carcinoma [45] melanoma [46]. GJA8 is amplified in Wilms tumors [47]. SEZ6L plays a role in signal transduction and protein-protein interaction and increases in lung cancer [48]. SLC5A1, a member of the GLUT family, encodes SGLT1 facilitating glucose transport at the basolateral membrane of the cells [49, 50]. Aberrant expression of SLC5A1 in different types of human cancers is observed, including ovarian cancer [51], cervical cancer [52], colorectal cancer [53, 54], hepatocellular carcinoma [55],prostate cancer [56]. SYNM is a type IV intermediate filament [57], which is supposed to modulate biological processes such as cell adhesion, cell motility. Reportedly, the silencing of synemin results in the suppression of tumor proliferation [58], whereas its hypomethylation is associated with aggressiveness in breast cancer [59]. Given all these data, the contradictory function of a single gene may ascribe to the “dual roles” of genes in different cancers, even in diversified stages of one cancer type.

Apart from predicting survival, the potential of prognostic classifiers lies in the ability to recognize patients that are more likely to respond to particular therapies [60]. Interestingly, in our study, the predictions of the prognostic risk model were significantly associated with the platinum response status, especially in the sensitive population, indicating the two models we built had a prominent correlation and may serve the clinical practice.

Finally, we screened out 3 hub genes, namely PNLDC1, SLC5A1 and SYNM, and explored their relationship with platinum therapy response and prognosis. The expression level of these genes detected by IHC in the FUSCC cohort indicates that PNLDC1 expresses higher in the resistant group, whereas overexpression of the SLC5A1 and SYNM were detected in the sensitive patients. Despite the insufficient number of events in OS analysis, all these 3 genes show significant correlations with PFS. Longer and more detailed follow-up data is required in further study. To date, the role of PNLDC1 and SYNM in OC has not been reported yet, and seldom do SLC5A1 as well, indicating they might be treated as new potential biomarkers in platinum-based chemotherapy and prognosis in OC, even as the therapeutic targets to some extent. For example, the development of individual SGLT1 inhibitors which target SLC5A1 is on the way [61].

Although our study brings new insights into OC treatment and survival, there are limitations to this study. Firstly, despite we included two completely independent datasets as training and validation cohorts and included an IHC cohort to validate our findings, this is a retrospective study. Next, the sample sizes in the training set, validation set, and IHC cohort were relatively small. Larger cohorts are required to prove our findings. Lastly, although bioinformatic analysis is a powerful tool for exploring potential biomarkers in tumors, further corroboration is needed.

## Conclusion

Taken together, based on the public databases, considering multiple aspects, we selected the feature DEGs to construct the SVM classifier to determine the patients’ responses to platinum-based chemotherapy. Meanwhile, a prognostic risk model was established to help predict patients’ prognoses. And 3 hub genes, PNLDC1, SLC5A1 and SYNM, related to the platinum therapy response and prognosis were screened out, which could be used as new biomarkers in OC treatment.

## Supplementary Information

**Additional file 1.**

**Additional file 2.**

**Additional file 3.**

**Additional file 4.**

**Additional file 5.**

**Additional file 6.**

**Additional file 7.** 

**Additional file 8.**

**Additional file 9.**

**Additional file 10.**

## Data Availability

The datasets used and/or analyzed in this study are available from the corresponding author on reasonable request.

## Abbreviations

OC: Ovarian cancer
TCGA: The Cancer Genome Atlas
GEO: Gene Expression Omnibus
SNP: Single nucleotide polymorphism
CNV: Copy number variation
NGS: Next-generation sequencing
DEGs: Differentially expressed genes
HRG: High-risk group
LRG: Low-risk group
PPV: Prediction value
NPV: Negative prediction value
AUC: Area under curves
ROC: Receiver operating characteristic curve
IOD: Integral optic density
PFS: Progression-free survival
MF: Molecular function
CC: Cellular component
BP: Biology procedure
GO: Gene Ontology
KEGG: Kyoto Encyclopedia of Genes and Genomes
RF: Random Forest
TMB: Tumor Mutation Burden
