# Supplementary figures and images for: A risk model of gene signatures for predicting platinum response and survival in ovarian cancer
Source: J Ovarian Res. 2022 Mar 31;15:39. doi: 10.1186/s13048-022-00969-3 (PMC8973612; doi:10.1186/s13048-022-00969-3)

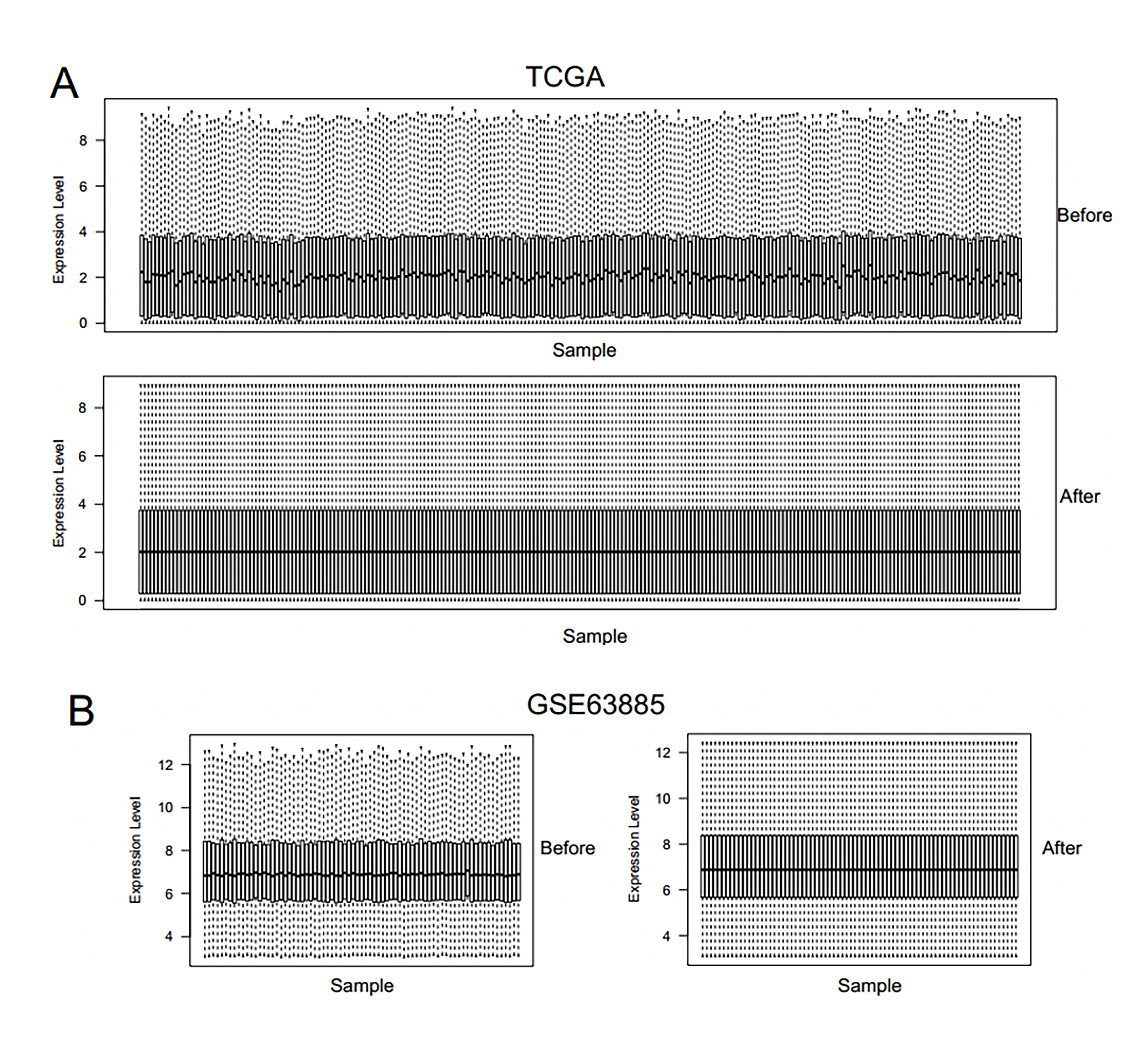

Supplement: Supplementary file 1 — Additional file 1. [file 13048_2022_969_MOESM1_ESM.png]
